# An information-theoretic foreshadowing of mathematicians’ sudden insights

**DOI:** 10.1073/pnas.2502791122

**Published:** 2025-08-18

**Authors:** Shadab Tabatabaeian, Artemisia O’bi, David Landy, Tyler Marghetis

**Affiliations:** ^a^Department of Cognitive and Information Sciences, University of California, Merced, CA 95343; ^b^Department of Psychological and Brain Studies, Indiana University, Bloomington, IN 47405-7007; ^c^Netflix, Department of Data and Insights, Los Gatos, CA 95032

**Keywords:** insight, complex systems, gesture, mathematical reasoning, early warning signals

## Abstract

The “eureka” insights that drive progress in science and mathematics remain shrouded in mystery. Sudden, unexpected, appearing like “flashes of lightning”, these insights have the hallmarks of critical transitions in complex systems. Here, zooming in on mathematicians working on proofs in their own departments, we show that sudden insights are anticipated by a system-agnostic, information-theoretic early warning signal. Using dense behavioral recordings of mathematicians’ moment-to-moment activity, we find that their blackboard interactions (e.g., writing, gesturing; N=4,653) became increasingly unpredictable before an insight, analogous to the critical fluctuations that anticipate transitions in physical and ecological systems. We explore analytically when this early warning signal applies to varied systems with discrete, symbolic dynamics. While bibliometric analyses offer a zoomed-out perspective on innovation, publications are a coarse-grained record of individuals’ insights. Explaining the sudden insights of innovators, from scientists to sculptors, requires attending to the local, distributed systems of their intellectual activity.

## Significance

The sudden “eureka” insights that drive progress in science and mathematics remain shrouded in mystery. Here, we borrow theory and methods from statistical physics and theoretical ecology to attempt to foreshadow the onset of these sudden insights. Using naturalistic video recordings of mathematicians working on proofs “in the wild” (i.e., in their own offices and seminar rooms), we show that insights that appear to come out of nowhere are actually prefigured by changes in how mathematicians are writing and gesturing. Our method for inferring when mathematicians are on the cusp of a breakthrough is quite general, so it may apply to other systems that generate a time series of discrete, symbolic events.

Mathematical insights involve sudden and unexpected transitions from confusion to clarity. Mathematicians describe these “aha!” or “eureka” insights as genuinely surprising, and afterward they are often unable to explain how they arrived at their insight (1). The polymath Henri Poincaré captured this in his description of an insight that arrived as he boarded a bus: “At the moment when I put my foot on the step, the idea came to me, without anything in my former thoughts seeming to have paved the way for it” (2, p. 388). The mathematician Carl Gauss described insight as “a sudden flash of lightning” and a “divine intervention” (1, p. 15). Such insights drive mathematical progress, yet their origins remain shrouded in mystery.

The suddenness of “aha” insights in mathematics is reminiscent of critical transitions—abrupt shifts from one stable regime to another—that occur in a variety of other complex systems (3, 4), from motor control (5) and mental health (6), to natural ecosystems and global climate (7). Critical transitions are sometimes anticipated by signals that the system is at risk of “tipping” into a new regime because the current regime is no longer stable (i.e., it is losing resilience) (4, 7–12). For instance, if a system gradually loses the ability to bounce back from perturbations (“critical slowing down”), then even small perturbations can push the system into an alternative regime, and the probability of a critical transition is thus high (8). Before a critical transition occurs, therefore, the increasing risk of tipping into a new regime can be revealed by so-called early warning signals or resilience indicators that reflect the system’s decreased capacity to respond to perturbations (e.g., “critical fluctuations”—increased variability in the system state) (4, 5, 7–9, 13–16). Such indicators have been found to anticipate critical transitions in a variety of systems, from physics and ecology (4, 8, 11, 12, 17), to human motor control, problem solving, mental health, and cognitive development (6, 9, 13, 18–21).

Could these early warning signals predict sudden insights in high-level mathematics? Mathematical activity occurs within a distributed system that often includes—in addition to a mathematician’s brain (22)—their body, a blackboard or notepad, and the inscriptions they create (23–29). This system has been compared to an ecosystem, with mathematicians actively constructing their own “notational niches” within which they can reason by inscribing (e.g., sketching, writing, erasing), gesturing (e.g., pointing to connect two inscriptions), and looking (e.g., shifting gaze from one inscription to another) (26, 30). If mathematical insights are critical transitions within this distributed system of mathematical activity, then they should be anticipated by signals that the system’s current regime is no longer stable or resilient.

Most existing early warning signals that a complex system is at risk of a critical transition, however, have been developed for systems where the overall state of the system can be captured by a few numerical variables (4, 8, 10), sometimes called order parameters, such as the population of an indicator species in a natural ecosystem (31), the relative phase of two moving body parts during coordinated movement (5), or the angular velocity of hand gestures while solving puzzles (19). Expert mathematical activity consists instead of sequences of events—writing an equation, pointing to a diagram, erasing a sketch, etc.—that are not easily summarized by a single order parameter. Most system-agnostic approaches to capturing a system’s loss of resilience, such as critical fluctuations, are thus ill-suited to the observable dynamics of mathematical activity. Moreover, signals of impending learning in elementary mathematics education, such as the mismatch between a student’s speech and gesture (32), are typically topic-specific (e.g., they require identifying specific types of gestures that are related to the lesson’s focal concept). Existing methods thus do not generalize to expert mathematical activity in which multiple mathematical techniques and ideas are combined flexibly and creatively.

Here, using an information-theoretic early warning signal, we show that sudden “aha!” insights among mathematical experts are anticipated by changes in the dynamics of their situated mathematical activity. Using video recordings of mathematicians working on proofs at blackboards in their own departments, we investigated whether we could anticipate the onset of sudden insights from subtle changes in their blackboard interactions. We reasoned that, since mathematical activity occurs within a distributed system that spans brain, body, and blackboard, a mathematician’s sudden insights should be preceded by signals within this distributed system. In particular, we sought to quantify the system’s loss of resilience—its openness to tipping into a new regime—from changes in mathematicians’ interactions with the blackboard. In what follows, we first describe our dataset of real-world proof activity by mathematical experts. We then introduce an information-theoretic measure that quantifies whether the system is at risk of tipping into a new regime, and illustrate our approach in a minimal dynamical model of mathematical insight. Finally, we show that this information-theoretic approach can anticipate mathematical insights in real-world expert reasoning.

## Documenting Blackboard Behavior and Sudden Insights in Expert Mathematics

**Fig. 1.**
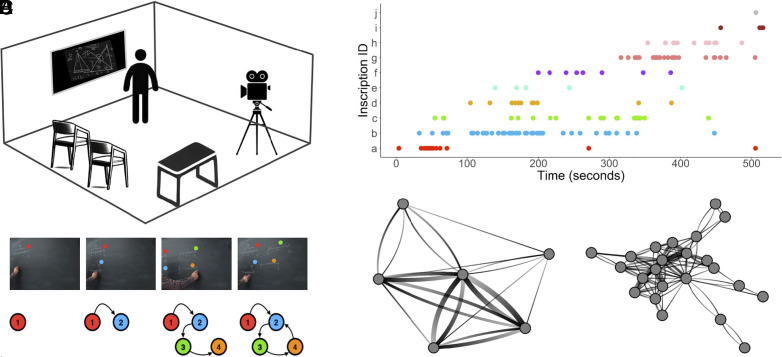
Studying mathematicians’ sudden insights in their natural habitat. (*A*) Illustration of the typical setting of mathematical reasoning in our video corpus. Mathematicians were video-recorded as they worked alone on proofs in their own departments (e.g., in their office or a seminar room). All mathematicians stood at a board, typically a chalkboard. (*B*) Moment-to-moment mathematical activity unfolded as a series of blackboard inscriptions and interactions. The *Top* row shows still images from a representative minute-long sequence of activity in which a mathematician created and interacted with four inscriptions (colored circles). Based on gesture, gaze, and speech, we identified whenever the mathematician overtly shifted their attention from one inscription to another. These shifts can be visualized as a directed network (*Bottom* row), with nodes representing inscriptions and edges representing shifts from one inscription to the next. The empirical probabilities of these transitions can be used to predict future behavior. (*C*) Time series of one mathematician’s interactions with inscriptions while working on a proof. Individual inscriptions are represented along the vertical axis and differentiated by color. Colored points indicate every moment when the mathematician shifted their attention to an inscription. (*D*) Mathematicians’ blackboard interactions varied considerably. Here, we visualize one mathematician’s blackboard interactions while working on two different proofs. Nodes represent inscriptions, edges represent shifts of attention from one inscription to the next, edge width represents the frequency of each shift, and edge shading indicates the direction of each shift (from light to dark).

To investigate sudden insights in expert mathematics, we video-recorded mathematicians while they worked alone at blackboards, chalk in hand, in their own departments (e.g., in their personal office; Fig. 1*A*), using an approach reminiscent of classic “think aloud” analyses of expert problem solving (33). The mathematicians were working on problems that we had selected from the William Lowell Putnam Mathematical Competition, an annual competition for undergraduate university students administered by the Mathematical Association of America. To convey a sense of the problems’ difficulty, this competition lasts six hours, consists of twelve problems, and in most years the median score is zero or one out of 120. To capture the moment-to-moment dynamics of the mathematicians’ situated mathematical activity, we identified each moment when they explicitly shifted their attention between inscriptions. We defined inscriptions as spatially adjacent and semantically related markings on the blackboard—for instance, a diagram in one corner of the board or an equation elsewhere (Fig. 1*B*). This annotation process generated a symbolic time series of thousands of discrete, moment-to-moment shifts in situated mathematical activity (N=4,653 shifts across 14 proof sessions). Fig. 1*C* visualizes this time series for one representative proof session. Mathematicians varied considerably in the number of inscriptions they introduced to prove a conjecture and in how they interacted with those inscriptions (Fig. 1*D*).

Within this video corpus, we identified every moment when mathematicians expressed in speech a sudden insight (e.g., “Oh I see!”). These moments were often accompanied by sudden movements and emotional exclamations, not unlike Archimedes running naked through the streets of ancient Syracuse yelling “eureka” (34), though admittedly less dramatic. Sudden insights were infrequent but occurred at least once in most proof sessions (N=27 insights across 13 of the 14 proof sessions). Presumably mathematicians also had other, perhaps smaller, insights that were not expressed verbally.

The question, then, is whether sudden “aha” insights are prefigured by changes in blackboard activity that may reflect a destabilization of the mathematician’s underlying understanding. In other complex systems, destabilization or loss of resilience is indexed by critical fluctuations (4, 5, 8, 9). Typically critical fluctuations are measured as the variance over time of some numerical variable (e.g., the population of an indicator species in an ecosystem). We reasoned that if an underlying continuous system were to drive observable behavior that is discrete and symbolic, then increased variability in the system’s underlying continuous dynamics should manifest as increased unpredictability of the discrete, symbolic behavior. Applying this reasoning to the case of situated mathematical activity, we predicted that the loss of resilience of a mathematician’s latent understanding should manifest as increasingly unpredictable blackboard interactions.

We formalize the notion of “unpredictable” behavior as the surprisal (a.k.a., information or self-entropy) (35, 36) of a shift from one inscription to another, given the mathematician’s recent behavior:

[1] $$\begin{array}{c} h\left(E_{t}\right)=-\log_{2}P\left(E_{t}|C_{t}\right) \end{array}$$

Here, $P(E_{t}|C_{t})$ is the probability of a behavioral event $E_{t}$ that occurred at time t, conditioned on recent blackboard interactions $C_{t}$. The value of surprisal, $h(E_{t})$, is equal to 0 when the event—that is, an attentional shift from one inscription to another—is exactly what you would expect to happen, based on recent behavioral context. Surprisal increases as an event becomes increasingly unusual (i.e., low probability) given recent behavioral context. [Cognitive scientists may recognize this measure from information-theoretic accounts of processing difficulty during language comprehension (37, 38).] We thus predicted that mathematicians’ sudden insights should be anticipated by the increased surprisal of their visible blackboard interactions.

## Information-Theoretic Early Warning Signals in a Minimal Model of Mathematical Insight

**Fig. 2.**
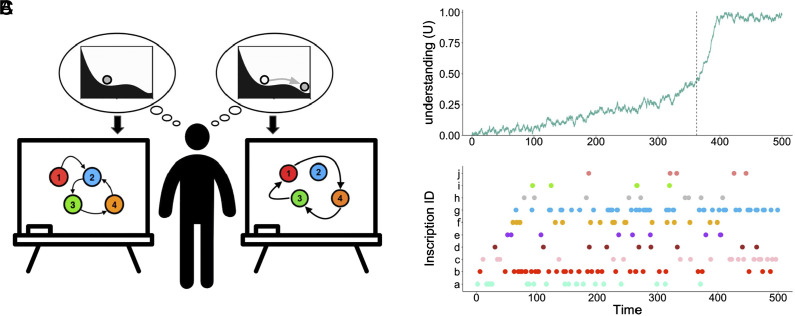
The challenge of inferring the stability of latent understanding from observable behavior. (*A*) Graphical illustration of a minimal model of how observable, discrete actions (e.g., pointing at a diagram, writing an equation) reflect latent, continuous understanding. Moment-to-moment understanding is not directly observable and consists of a continuous trajectory in a space of ideas (illustrated by the gray balls in the potential landscapes in the thought bubbles). Latent understanding drives observable actions, with the current state of understanding determining the probability of transitioning from interacting with one inscription to the next (illustrated by the transition networks on the blackboards). A single control parameter, γ, determines the relative influence of innovation versus functional fixedness. For smaller values of γ, the dynamics of understanding are dominated by misconception; for larger values of γ, shown in both thought bubbles, the insight attractor dominates. The transition from confusion (*Left*) to enlightenment (*Right*) can be sudden, with understanding tipping from one basin of attraction to another. We implemented this framework as a minimal mathematical model in which the dynamics of latent understanding are governed by a stochastic differential equation, and latent understanding determines the transition probabilities of a Markov process that generates discrete, observable interactions with inscriptions (see *Materials and Methods* for details). (*B*) The dynamics of latent understanding from a representative run of the minimal model, in which the control parameter γ increases linearly with time. When gamma passes a critical value (γ=0.26, vertical dashed line), the “confused” attractor disappears. In each simulated proof session, latent understanding remains low at first but undergoes a critical transition, with a rapid increase from confusion (U<0.5) to enlightenment (U≈1). (*C*) Interactions with inscriptions from the same run of the minimal model shown in (*B*). Latent understanding determines the probabilities of transitioning from each inscription to another. Individual inscriptions are represented along the vertical axis and differentiated by color. Colored points indicate every moment when the simulated mathematician shifted their attention to an inscription. Anticipating a sudden insight from mathematical behavior involves inferring the stability of underlying understanding (shown in panel *B*) from the dynamics of observable behavior (shown in panel *C*).

To illustrate our approach, we describe a minimal model of insight in which latent understanding drives observable behavior in the form of discrete events. We assume that the dynamics of understanding consist of a trajectory through a continuous state space (39, 40). This minimal model is meant to capture key, qualitative features of the experience of insight. First, the model captures the stubbornness of initial misconceptions, which tend to resist change. Second, it formalizes the tension between this stubbornness and the desire for improved understanding. Third, it explains the rapid, unexpected transition from confusion to enlightenment that is characteristic of insight. And fourth, it conveys the distinction between the continuous dynamics of latent understanding and the discrete, symbolic dynamics of blackboard activity (Fig. 2).

We assume for simplicity that understanding varies along a single dimension, U, that can range continuously from 0 (total misconception) to 1 (perfect understanding). An insight, then, is a sudden increase in U. Assuming that an individual begins in a state of misconception, the dynamics of U reflect two nonlinear processes: functional fixedness, F, the tendency to persist in one’s initial conception (41), which pushes understanding back toward 0, and innovation, I, which increases understanding, pushing it toward 1. The relative contribution of innovation and functional fixedness is set by a control parameter, γ:

[2] $$\begin{array}{c} \frac{\mathrm{dU}}{\mathrm{dt}}=\gamma I-F. \end{array}$$

We assume that functional fixedness follows an inverted U-shaped curve, initially increasing as understanding departs from an initial conception but then dropping off as a new understanding is approached. We assume that the influence of innovation is greatest when understanding is far from the correct insight and decreases as understanding approaches the correct insight. To capture these dynamics, we adapted the functional form of a well-studied model of metastable dynamics in ecology (42, 43), implemented as a stochastic differential equation, so that the dynamics of understanding U were driven by functional fixedness, F, innovation, I, and a small noise term that captures the mathematician’s uncertainty (*Materials and Methods*).

In this model, the tension between functional fixedness and innovation generates dynamics of understanding with two attractors: a “confused” regime with U closer to 0 and an “enlightened” regime with U closer to 1 (Fig. 2 *A* and *B*). For a range of intermediate values of the control parameter γ, both these regimes exist and are resilient (*SI Appendix*, Fig. S1); thus, if understanding begins in the “confused” regime, then that initial confusion will persist. But as γ increases, the drive to innovate comes to dominate the dynamics of understanding, and the resilience of the “confused” state decreases (*SI Appendix*, Fig. S1*C*). Eventually, γ reaches a critical value at which the “confused” attractor disappears and a critical transition occurs, with understanding increasing rapidly toward the correct conception—that is, a sudden insight occurs (Fig. 2*A*).

In the case of real-world mathematical activity, we do not have direct access to mathematicians’ latent understanding. What we can observe are overt behaviors. The model assumes that these behaviors consist of discrete actions (e.g., writing an equation, pointing to a variable, etc.), and that the probability of an action is determined by the mathematician’s latent understanding (Fig. 2*C*). As latent understanding changes, so does the probability of each action (see *Materials and Methods* for details). As an illustration, consider a mathematician who thinks that two equations are unrelated; they are unlikely to shift their attention directly from one to the other. But if they understand a sketch as offering a visualization of a function, then they are likely to shift their attention from one to the other.

To be clear, this model of coupled latent understanding and overt behavior is not intended to be a psychologically realistic process-model of insight. It is a minimal model that shows formally how a few key elements can interact to generate essential qualitative features of mathematical insight (44, 45). We use it here to illustrate how our information-theoretic approach can infer the destabilization of underlying continuous dynamics from changing patterns of discrete, symbolic observations.

To that end, we used this model to generate synthetic proof sessions consisting of a time series of latent, continuous understanding and a coupled time series of observable, discrete activity (Fig. 2 *B* and *C*). For each synthetic proof session, latent understanding started low, in the “confused” regime. The control parameter, γ, increased gradually until it passed the critical value at which the “confused” regime disappears; around this time, understanding increased rapidly as it entered the “enlightened” regime. In each synthetic proof session, therefore, mathematicians began in confusion and eventually had a sudden insight (Fig. 2*B*).

**Fig. 3.**
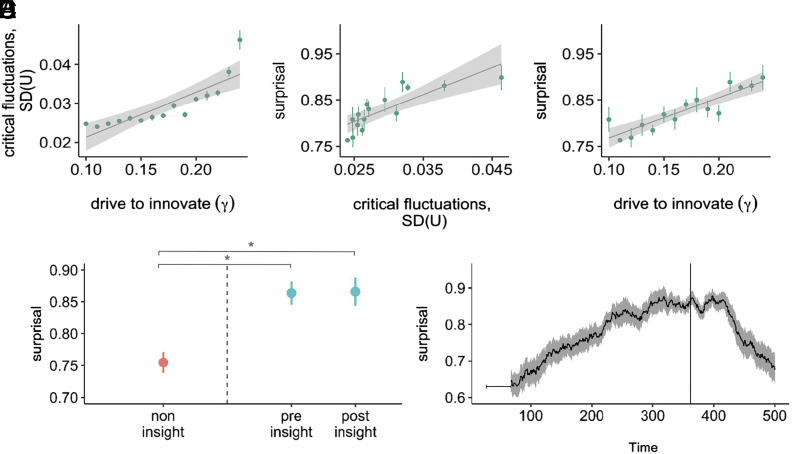
Anticipating sudden insights in a minimal model of mathematical insight. (*A*) In the model, as the control parameter γ approaches the critical value at which the “confused” attractor disappears (γ=0.26), latent understanding becomes more variable. These critical fluctuations are thus a signal that the “confused” attractor is losing resilience. Points show means from 24 simulation runs, and error bars show SEs. The line shows a linear fit with a 95% CI. (*B*) In the model, an increase in the critical fluctuations of latent, continuous understanding is associated with an increase in the unpredictability of observable, discrete interactions. (*C*) In the model, as the control parameter γ approaches the critical value at which the “confused” attractor disappears (γ=0.26), discrete, observable interactions become more unpredictable. The unpredictability of observable, discrete interactions is thus an information-theoretic signal that the latent “confused” attractor is losing resilience. (*D*) In the model, interactions were significantly more unpredictable in the vicinity of an insight. Observable, discrete interactions that occurred when γ was far from the critical value (red) were more predictable than interactions that occurred immediately before and immediately after γ surpassed the critical value and latent understanding underwent a critical transition from confusion to enlightenment. (Dots indicate means across simulation runs, N=24; error bars indicate SEs; * indicates P<0.001.) (*E*) In the model, observable interactions became increasingly unpredictable in the lead-up to a sudden insight. The solid vertical line represents the moment of insight (i.e., the critical transition) in the model. The thick black line indicates mean surprisal in a sliding window; the ribbon indicates SE; the initial gray line indicates the width of the sliding window.

As predicted, in the model these insights were anticipated by critical fluctuations in the continuous dynamics of latent understanding. As γ approached the critical threshold, latent understanding U remained on average quite low. Based only on mean values of U one might not suspect that a sudden, qualitative change in understanding was on the horizon. However, the dynamics of latent understanding exhibited critical slowing down, a dampened response to perturbations. As a consequence, “sudden” critical transitions from confusion to insight were anticipated by critical fluctuations in latent understanding (i.e., increasing variability), in line with past findings with similar dynamical models (42, 43). As γ increased and the “confused” regime lost resilience, critical fluctuations in latent understanding increased systematically (Kendall’s rank correlation between γ and SD of U: τ=0.943, T=102, P<0.001) (Fig. 3*A*).

In real-world mathematical activity, however, we do not have access to mathematicians’ latent understanding, just their observable behavior. We thus analyzed, in the model, whether the loss of resilience of the “confused” regime would manifest as increasingly unpredictable dynamics of their discrete, symbolic behavior. As predicted, increased critical fluctuations in latent understanding caused observable mathematical behavior to become increasingly unpredictable (Kendall’s rank correlation between critical fluctuations in latent understanding and surprisal of observable behavior: τ=0.771, T=93, P<0.001, Fig. 3*B*). Sudden insights in the model were thus anticipated by an increase in the surprisal of observable behavior, with a gradual increase in surprisal as γ increased and the system lost resilience (τ=0.752, T=92, P<0.001; Fig. 3*C*).

In this minimal model, observable mathematical activity was significantly more unpredictable before an insight (M=0.86, SE=0.01) and remained unpredictable in the period immediately afterward (M=0.86, SE=0.02), compared to activity at other times (M=0.75, SE=0.01). A linear mixed effects model confirmed that surprisal was significantly greater immediately before the moment of insight (b=0.098±0.02SE, t=3.52, P<0.001) and immediately after the moment of insight (b=0.095±0.02SE, t=3.31, P<0.001) (Fig. 3*D*). This reflected a gradual ramping up of behavioral unpredictability as the moment of insight approached, peaking near the moment of insight (Fig. 3*E*). Thus, in a minimal model of mathematical activity, the resilience of latent, continuous understanding was indexed by the predictability of the observable, discrete behavior.

In *SI Appendix*, we explore analytically when this result holds in general. If a real-valued variable governs the dynamics of a symbolic process, under minimal assumptions about the mapping from the latent real-valued variable to the symbolic process, increased variability in the latent numerical variable will often manifest as more unpredictable (i.e., higher surprisal) dynamics in the symbolic time series. As a consequence, if a complex system’s unobservable order parameter exhibits critical fluctuations in the lead up to a critical transition, then the system’s observed symbolic dynamics can often be used to infer the system’s resilience.

## Anticipating Sudden Insights in Real-World Mathematical Activity

**Fig. 4.**
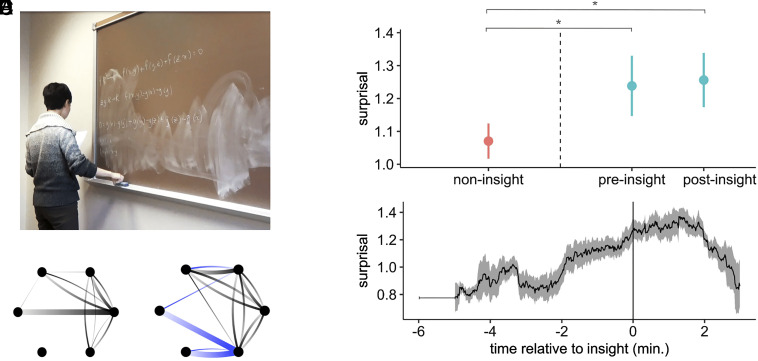
Mathematicians’ sudden insights were anticipated by the increased unpredictability of their blackboard interactions. (*A*) The moment of insight of one mathematician, marked by verbal expression of “aha!.” (*B* and *C*) Blackboard interactions before and after the moment of insight shown in (*A*). Nodes represent blackboard inscriptions; edge width indicates the frequency of a shift of attention from one inscription to another, directed along the gradient (from light to dark). Prior to the insight (*B*), some inscriptions were never connected by the mathematician. During the insight (*C*), the mathematician’s activity connected inscriptions that previously were never connected (blue edges) and no longer connected inscriptions that had been previously [e.g., the thick black edge running horizontally in (*B*) that disappears in (*C*)]. (*D*) In real mathematicians, interactions were significantly more unpredictable in the vicinity of an insight. Observable blackboard interactions that occurred immediately before and after a sudden insight (teal) were more unpredictable than interactions that occurred at other times (red) across all proof sessions in the corpus. (Dots indicate means; error bars indicate SEs; * indicates P<0.01.) Compare to the model results in Fig. 3*D*. (*E*) In real mathematicians, across all proof sessions in the corpus, blackboard interactions became increasingly unpredictable in the lead-up to a sudden insight. Time is rescaled so that 0 is the moment of insight (solid vertical line). The thick black line indicates mean surprisal in a sliding window; the ribbon indicates SE; the initial gray line indicates the width of the sliding window. Compare to the model results in Fig. 3*E*.

We next asked whether this information-theoretic measure could anticipate the approach of sudden insights in real-world mathematical activity. We looked at blackboard interactions in the periods around moments of sudden insight. The mathematician in Fig. 4*A*, for instance, has suddenly exclaimed “aha,” and this was accompanied by a change in their behavior (Fig. 4 *B* and *C*). Around the period of insight, they abandoned connections that were previously common, no longer shifting their attention between objects in ways they had previously (e.g., the thick horizontal edge in Fig. 4*B* that is absent in Fig. 4*C*). Instead, this mathematician began to connect previously disconnected inscriptions, shifting their attention from one inscription to another in ways that had not occurred previously (blue edges in Fig. 4*C*). In this case, therefore, the insight was associated with blackboard activity that differed from past behavior.

More concretely, consider the behavior of another mathematician in the corpus who experienced three sudden insights while attempting to prove a conjecture. Early on, she drew a line on the blackboard to represent a bounded interval of real numbers. Whenever her attention was directed toward this line, she most frequently transitioned her attention next to a nearby list of real numbers. As a result of how common this transition was, its surprisal was typically quite low (<h>=1.2, corresponding to a conditional probability of P=0.44, given her recent behavior) and never greater than h=2.6 (i.e., a conditional probability of P=0.17, given her recent behavior). These low-surprisal transitions were expected, familiar moves, within the context of her recent blackboard interactions. In contrast, this mathematician seldom shifted her attention from the line representing a bounded interval to a drawing of a triangle on the opposite side of the blackboard—but halfway through the proof session, approximately thirty seconds before experiencing a sudden insight, she made exactly this transition, with an associated surprisal of h=3.16 (i.e., a conditional probability of only P=0.11, given her recent behavior). In the lead-up to a sudden insight, therefore, she abandoned a pattern of blackboard interaction to explore a new connection.

This was true in general across all insights in the corpus. We analyzed surprisal with a linear mixed-effects model (see *Materials and Methods* for details). Overall, the unpredictability of situated mathematical activity did not change over the course of each session (change in surprisal with each passing minute: b=0.003±0.002SE, t=1.622, P=0.1). However, situated activity was significantly less predictable when an insight was nearby (Fig. 4*D*). Mathematicians’ situated activity was more unpredictable in the periods surrounding an insight, both in the period immediately before the insight (M=1.23, SE=0.09; b=0.158±0.05SE, t=2.83, P<0.01) and in the period immediately after (M=1.25, SE=0.08; b=0.209±0.05SE, t=3.871, P<0.01), compared to their activity at other times (mean surprisal: M=1.07, SE=0.05). We confirmed the robustness of this effect with a time-permuted null model. We generated a time-permuted surrogate time series for the entire corpus by randomly shuffling the time of blackboard interactions within each proof session. This preserves the number of inscriptions, the number of interactions with each inscription, and the timing of when mathematicians interacted with inscriptions, but randomizes which inscription is interacted with at each moment. We repeated this process (N=500) and for each surrogate time series we calculated the increase in surprisal before and after insights. The empirically observed increase in surprisal was reliably greater than expected under this null, both before (P=0.03) and after (P<0.01) sudden insights (*SI Appendix*, Fig. S4).

This process of behavioral destabilization began minutes before the insight occurred (Fig. 4*E*). Mathematicians’ situated activity became increasingly unpredictable in the lead up to a sudden insight, starting more than two minutes before they expressed an insight (Fig. 4*E*). Surprisal ramped up gradually in the minutes before the insight, peaked after the moment of insight, and then decreased precipitously. This drop in surprisal after the insight suggests that mathematicians settled into a new regime, as their blackboard interactions were now more predictable from their recent postinsight behavior. Thus, as predicted by our minimal model of mathematical insight (Fig. 3), real-world insights among mathematicians were anticipated by a gradual loss of the predictability of their situated activity (Fig. 4).

## Discussion

The suddenness of mathematical insight lends it an air of mystery. Here, we showed that some mathematical insights that appear suddenly—like a “flash of lightning,” in Gauss’s phrase—do not arrive without warning but instead are prefigured by changing patterns of blackboard interaction. In the minutes leading up to an insight, mathematicians’ shifts of attention between inscriptions enacted connections that were increasingly unprecedented. This kind of associative or combinatorial thinking is a hallmark of creativity (46–50), including in scientific arenas such as biomedical research (51) and technological innovation (52). The mathematician Henri Poincaré went so far as to describe his insights as the recognition of an “unsuspected kinship between other facts, long known, but wrongly believed to be strangers to one another” (2). We captured this combinatorial process as it unfolded moment-to-moment in mathematicians’ embodied activity. After an insight, however, comes the hard work of verification (53). The critical transitions that we identified here in the “context of discovery” may be complemented by critical transitions in confidence in the “context of justification” (54) as mathematicians work out the inferential details of a proof (55).

Our approach combines the use of computational methods with a focus on the microdynamics of discovery. It thus shares features both with “science of science” that zooms out to bibliometric data to quantify the long-timescale dynamics of entire careers or disciplines (51, 52, 56), and with ethnographic or qualitative studies of scientists and mathematicians that have offered accounts of their local, situated practices (25, 27, 57, 58). Our approach blends features of both traditions, along with data that are much more fine-grained than are typically available on expert reasoning, to direct a computational “microscope” on situated activity in an ecologically valid setting. This approach is not limited to the study of mathematics, and our information-theoretic early warning signal should generalize to many symbolic practices, complementing other information-theoretic approaches that have been developed for continuous systems or more elementary problem solving (18, 19). Similar methods could be deployed to investigate insight and understanding in other scientific disciplines, from the biologist’s bench to the theoretical physicist’s blackboard, and even in artistic domains such as painting or sculpture. One exciting possibility is that fine-grained analyses of situated activity, as reported here, could be combined with analyses of long-timescale innovation, as captured by bibliometric data, to generate insight into the nested timescales of creativity. Such hybrid datasets could even be sufficiently fine-grained to analyze using methods that predict not just the onset but also the outcome of a critical transition (11, 12)—in other words, to forecast not just when an insight is about to occur but what that insight is likely to be.

A variety of factors might explain the gradual increase in unpredictable activity in the lead up to mathematical insight. Some might be implicit or subconscious. A period of prolonged intellectual impasse with little progress, for instance, might exacerbate a mathematician’s frustration, implicitly reducing their conceptual inertia and increasing their openness to innovation (59). Or a prolonged impasse might decrease the salience of initial ideas, allowing other ideas to come to the fore in subconscious reasoning (53). The increase in unpredictable activity could also reflect conscious choices. Mathematicians might decide, explicitly, to make a tactical switch from exploiting an initial conception to exploring for new ones. Strategically trading off between exploration and exploitation while “foraging” for ideas has been implicated in human reasoning and creativity across timescales (60), from short-term problem solving (61), to scientific and artistic “hot streaks” (62), to Darwin’s lifelong reading habits (63). Since scientific contributions that involve surprising connections have greater impact (64), and advice books on mathematical problem solving recommend explicitly to “recombine [a problem’s] elements in some new manner” (65), experts in search of insight might decide intentionally to seek out novel connections. Increasingly unexpected blackboard interactions might also reflect a shift toward a style of curiosity in which the mathematician “experiments and breaks with traditional pathways of investigation” (66). At a biological level, this increase in behavioral unpredictability might reflect a destabilization of underlying brain dynamics. In humans, for instance, unstable neural activity can precede a sudden behavioral transition (15), and research on rodents has found that belief change is accompanied by increased variability in neural activity (16). Future work could investigate whether sudden mathematical insights are foreshadowed by the destabilization of brain areas thought to be specialized for mathematical thought (22, 67).

Alternatively, mathematical insights might be driven by the blackboard interactions themselves, in line with accounts of intelligence that adopt a unit of analysis larger than the brain alone (30, 68–70). Mathematical activity reliably spans brain, body, and blackboard (23–29, 33, 71, 72). Mathematicians themselves take these interactions seriously: Despite hypoallergenic whiteboards and even digital alternatives, old-fashioned chalk and blackboard remain central to mathematical practice (25, 73, 74), with some mathematicians even obsessing over the perfect chalk (75). Inscription is at the core of mathematical reasoning. Even slight modifications to notations can influence mathematical performance (71, 76). And once ideas are externalized as inscriptions, they become easy to connect—not by laboriously bringing them together in working memory, but by linking them in the external world with gestures (29, 70, 77, 78) or movements of the eyes (79). Old ideas can be rediscovered with a single glance. New connections can be discovered by accident as one’s hands and eyes are drawn across the blackboard. The entire distributed system of situated activity may be the engine of mathematical insight.

## Materials and Methods

### Naturalistic Video Corpus of Mathematicians.

We leveraged a unique dataset of naturalistic recordings of PhD-level mathematicians working in their own departments, either in their own office or in a shared seminar room. Participants gave informed consent to a protocol approved by Indiana University’s Institutional Review Board (protocol #1505609910). For approximately one hour, mathematicians attempted to prove conjectures from the William Lowell Putnam Mathematical Competition, an annual competition sponsored by the Mathematical Association of America. (We provide the conjectures in *SI Appendix*.) This dataset contains thousands of discrete interactions—pointing, writing, erasing—occurring over multiple hours of real-world activity (see *SI Appendix* for details). Our analyses here focused on mathematicians who had each worked on two conjectures, one set theoretic and the other geometric (N=6 mathematicians, 3 men, and 3 women). These 12 proof sessions lasted a total 4 h and 5 min. Results are unchanged when we include a pilot session with an additional mathematician (*SI Appendix*, Fig. S2).

One researcher, unaware of our hypotheses about mathematical insight, coded from the video every time a mathematician explicitly directed their attention toward a mathematical inscription, whether by creating a new inscription or by shifting their attention to an existing inscription. An inscription was defined as spatially proximal and semantically related markings, such as an entire equation or the entire plot of a function. Shifts of attention (N=4,653) were inferred from mathematicians’ gaze, gesture, speech, writing, or the act of erasing an inscription. A second coder identified from the video every time a mathematician expressed in speech that they were having an insight (e.g., “aha” or “ohhhhhh, I see;” N=24 across 12 proof sessions; or 27 when including a pilot session as reported in the *SI Appendix*).

### Information-Theoretic Measure of Loss of Resilience in a Symbolic Time Series.

To quantify the unpredictability of mathematicians’ blackboard activity, we used surprisal, an information-theoretic measure of how unexpected or informative an event is. We calculated the surprisal of each shift of attention relative to shifts that had occurred in the recent past:

[3] $$\begin{array}{c} h\left(E_{t}\right)=-\log_{2}P\left(E_{t}|C_{t}\right) \end{array}$$

Here, $P(E_{t}|C_{t})$ is the probability of a behavioral event $E_{t}$ at time t, conditioned on recent blackboard interactions $C_{t}$. We calculated the empirical transition probabilities between inscriptions in the context $C_{t}$, which were then used to calculate the surprisal of the new event $E_{t}$. Surprisal, $h(E_{t})$, is equal to 0 when the event is perfectly predicted by recent behavioral context (i.e., probability of 1), and increases as an event becomes increasingly unusual (i.e., low probability) compared to recent behavioral context. We limited the context, $C_{t}$, at time t to behavior in a window of fixed duration δ immediately preceding the event ($C=\{E_{c}\}$, for t−δ<c≤t). The context $C_{t}$ is thus the ordered sequence of events that occurred in the window of duration δ that ends at time t. For empirical analyses, we used a 60-s context window; for minimal model analyses, we used a 40 time-step context window. A sensitivity analysis of the context width δ for values ±33% of the value used in the Main Text confirmed that results are robust to this parameter choice (*SI Appendix*, Fig. S3).

### Minimal Model of Situated Insight.

The minimal model of mathematical insight generates continuous dynamics of latent understanding, which drive the symbolic dynamics of observable behavior. The dynamics of understanding U reflect two pressures. The first, functional fixedness, is the tendency to revert to an initial misconception. The second, innovation, is a drive to improve one’s understanding. Functional fixedness initially increases as understanding departs from an initial conception and then decreases as a correct understanding is approached. Innovation is a monotonically decreasing function of U, greatest when understanding is far from the correct insight and decreasing as understanding approaches the correct insight. We modeled the continuous dynamics of understanding, U, as a stochastic differential equation, with a functional form adapted from ecology (42, 43):

[4] $$\begin{array}{c} \frac{\mathrm{dU}}{\mathrm{dt}}=\gamma \frac{{\left(1-U\right)}^{2}}{\left(0.1^{2}+{\left(1-U\right)}^{2}\right)}-U\left(1-U\right) \end{array}$$

The first term reflects the tendency to innovate. The second term reflects functional fixedness. The relative contribution of innovation is determined by the control parameter, γ. We chose this functional form because it resembles a well-studied model of metastable dynamics (42, 43) that generates some of the qualitative features of sudden insight. We imposed a regime of stochastic forcing, so that U is perturbed by infinitesimal “kicks” due to stationary Gaussian white noise (see *SI Appendix* for details).

In the model, observable behavior is a symbolic Markov process, with the probability of shifting attention from one inscription to another determined by current understanding $U_{t}$. Each run was initialized with two random transition matrices, one that corresponded to complete confusion ($B_{0}$) and one that corresponded to enlightenment ($B_{1}$). The transition probabilities governing observable behavior for intermediate values of understanding were linearly interpolated between these extremes: $B_{U}=\left(1-U\right)*B_{0}+U*B_{1}$.

To correspond with the empirical dataset, we simulated N=24 synthetic proof sessions, each with N=25 inscriptions. Stochastic dynamics were simulated using Euler’s method (80), with a Weiner process noise term (i.e., increments drawn independently from a standard Normal distribution). On each run, the control parameter γ increased linearly over time from 0.1 to 0.3. Both “confused” and “enlightened” attractors exist for 0.1<γ<0.26, but the “confused” attractor disappears at the critical value γ=0.26 (*SI Appendix*, Fig. S1 *A*–*D*).

As we show in *SI Appendix*, our results are quite general and not specific to this particular implementation or functional form. Under a few minimal assumptions about the relation between the latent continuous dynamics and the observable symbolic process, increasing surprisal of the symbolic process is a reliable index of increased fluctuations in the latent continuous dynamics.

### Statistical Analyses.

All analyses were conducted in R using the lmerTest package. Analyses of the empirical data in the Main Text exclude an initial pilot session; results were qualitatively unchanged when we included this pilot session (*SI Appendix*). For both the model and the empirical data, we analyzed the temporal dynamics of surprisal using multilevel linear models that accounted for between-session random variability. These models included fixed effects for time, whether the event was in the period before an insight, whether the event was in the period after an insight, and whether the event involved introducing a new inscription. Full model specifications are available in *SI Appendix*.

All data and analysis scripts are publicly available on the Open Science Framework: https://osf.io/fnepw/?view_only=b6606826e5214189b100d456e571511d.

## Supplementary Material

Appendix 01 (PDF)

## Data Availability

Anonymized CSV file data have been deposited in Open Science Framework (https://osf.io/fnepw/?view_only=b6606826e5214189b100d456e571511d) (81).
