# Supplementary material for: An information-theoretic foreshadowing of mathematicians’ sudden insights
Source: Proc Natl Acad Sci U S A. 2025 Aug 18;122(35):e2502791122. doi: 10.1073/pnas.2502791122 (PMC12415256; doi:10.1073/pnas.2502791122)
Supplement: Supplementary file 1 — Appendix 01 (PDF) [file pnas.2502791122.sapp.pdf]

Supporting Information:
An information-theoretic foreshadowing of mathematicians' sudden insights

Shadab Tabatabaeian[1,*], Artemisia O'bi , David Landy[2, 3], and Tyler Marghetis[1,*]

[1]University of California, Merced
[2]Netflix
[3]Indiana University, Bloomington
[*]corresponding authors: stabatabaeian@ucmerced.edu and tyler.marghetis@gmail.com

Contents

1 Supplementary Materials and Methods **1**
1.1 Naturalistic corpus of mathematicians . . . 1
Corpus creation • Problems selected from the William Lowell Putnam Mathematical Competition • Annotation of the Mathematics Experts Video Corpus
1.2 Analyses . . . 2
Information-theoretic measure of loss of resilience in a symbolic time series • Statistical models
1.3 Minimal formal model of situated insight . . . 3
2 Supplemental Results **4**
2.1 Coupled real-valued and symbolic dynamics: Analytic results . . . 4
Setup • Argument
2.2 Empirical results with the addition of pilot proof sessions . . . 6
2.3 Window size sensitivity analysis for the empirical results . . . 7
3 Supplemental Figures **8**

1 Supplementary Materials and Methods

1.1 Naturalistic corpus of mathematicians

1.1.1 Corpus creation

We leveraged a dataset collected to study mathematical reasoning as it occurs in its natural context: in the Math department, standing at a blackboard, with chalk in hand. The dataset consists of naturalistic recordings of mathematicians working in their own departments, either in their own office or in a shared seminar room. The mathematicians were completing their PhD in mathematics at an R1 research university. Each mathematician worked for up to an hour, moving on to a new conjecture when they either felt they had proved the current conjecture or did not know how to make further progress. The mathematicians were encouraged to verbalize their reasoning while working on the proof, but otherwise were left to their own devices [1].

For approximately one hour, each mathematician attempted to prove conjectures from the William Lowell Putnam Mathematical Competition, an annual competition sponsored by the Mathematical Association of America. (We provide the conjectures below.) This dataset contains thousands of discrete interactions

— pointing, writing, erasing — occurring over multiple hours of real-world activity. Our analyses here focused on mathematicians who had each worked on two conjectures, one set theoretic and the other geometric ($N = 6$ mathematicians, 3 men and 3 women). These 12 proof sessions lasted a total 4 hours and 5 minutes. Results are unchanged when we include a pilot session with an additional mathematician (Supplementary Fig. S2).

The subset of the video corpus that we analyzed in the Main Text included six mathematicians working on conjectures 2 and 3 for a total of 4 hours and 5 minutes; in the Supplemental Results below, we show that the results are unchanged when an additional pilot mathematician is included in the dataset.

1.1.2 Problems selected from the William Lowell Putnam Mathematical Competition

Mathematicians in the video corpus were working on conjectures selected from the annual William Lowell Putnam Mathematical Competition. The problems were chosen to be non-trivial but approachable for PhD-level mathematical experts. They touched on various mathematical subfields, including set theory, geometry, and analysis:

1. Find an uncountable subset, S, of the power set of a countable set, such that the intersection of each pair of elements in S is finite.
2. Let f : R2→R be a function such that f(x, y) + f(y, z) + f(z, x) = 0 for all real numbers x, y, and z. Prove that there exists a function g: R→R such that f(x, y) = g(x) - g(y) for all real numbers x and y.
3. Let $d_1, d_2, \ldots d_{12}$ be real numbers in the interval $(1, 12)$. Show that there exist distinct indices i, j, k such that d_i, d_j, d_k are the side lengths of an acute triangle.

1.1.3 Annotation of the Mathematics Experts Video Corpus

The video was annotated on a frame-by-frame basis, using ELAN video annotation software [2], by identifying and coding every time a mathematician directed their attention at a blackboard inscription. This could occur by creating a new inscription or shifting attention to an existing one. Shifts of attention were inferred from mathematicians' gaze, gesture, speech, writing, or erasing. This generated a time series of "events" in which mathematicians' shifted their attention among inscriptions. Distinct inscriptions were identified based on two criteria: semantic relatedness and spatial proximity. For example, the different parts of a graph — e.g., two axes, axes labels, the line representing the function — would be treated as a single inscription. All coding was conducted in ELAN, software for annotating audio and video [3].

A second coder identified, from the video, every time that a mathematician verbally expressed that they were having an insight (e.g., saying "aha!" or "ohhhhhh, I see"). A total of 27 insights were identified in the corpus (including the proof sessions of the pilot mathematician). Both coders were blind to our hypothesis about mathematical insights.

1.2 Analyses

1.2.1 Information-theoretic measure of loss of resilience in a symbolic time series

To quantify the unpredictability of mathematicians' blackboard activity, we used surprisal, an information-theoretic measure of how unexpected or informative an event is. We calculated the surprisal of each shift of attention relative to shifts that had occurred in the recent past:

$$h(E_t) = -\log_2 P(E_t | C_t) \tag{1}$$

Here, $P(E_t|C_t)$ is the probability of a behavioral event E_t at time t, conditioned on recent blackboard interactions C_t. We calculated the empirical transition probabilities between inscriptions in the context C_t, which were then used to calculate the surprisal of the new event E_t. Surprisal, $h(E_t)$, is equal to 0 when the event is perfectly predicted by recent behavioral context (i.e., probability of 1), and increases as an event becomes increasingly unusual (i.e., low probability) compared to recent behavioral context. We limited context to behavior in a window of fixed duration δ immediately preceding the event ($C = \{E_c\}_{(t-\delta)<c<t}$). For empirical analyses, we defined context as the 60-second window preceding an event; for model analyses, we used a 40-step window. Sensitivity analyses confirmed that results were robust to variation in these analytic parameters.

1.2.2 Statistical models

All analyses were conducted in `R` using the `lmerTest` package. Analyses of the empirical data in the Main Text exclude an initial pilot session; results were qualitatively unchanged when we included this pilot session (see Supplementary Results). For both the model and the empirical data, we analyzed the temporal dynamics of surprisal using multilevel linear models that accounted for between-session random variability. These models included fixed effects for time, whether the event was in the period before an insight, whether the event was in the period after an insight, and whether the event involved introducing a new inscription. Here we specify the regression models reported in the Main Text and the Supplemental Materials, using the syntax of the R statistical programming language [4]:

```
surprisal ~ 1 + event type (attention or introduction) + isBeforeInsight
* isAfterInsight + time + (1|proof session)
```

This is a generalized linear mixed-effects model of surprisal, with fixed effects for time, the temporal relation of attentional shifts to insight (specifically, whether they occurred before or after insight), and whether the event involved introducing a new inscription. Random intercepts were added for each proof session to account for individual differences. All models were fit using the `lmerTest` package [5].

Previous work has suggested that the insight process can begin prior to its verbal expression (the "flash" of insight) and can continue for some time after [6]. We thus analyzed the periods immediately before and after the moment of the verbal expression of the insight. Inspection of the video corpus suggested that one-minute periods before and after the insight would capture the pace at which mathematicians progressed through their reasoning. A sensitivity analysis confirmed that our results do not depend on the choice of this specific duration.

1.3 Minimal formal model of situated insight

The minimal model of mathematical insight generates continuous dynamics of latent understanding, which drive the symbolic dynamics of observable behavior. The dynamics of understanding U reflect two pressures. The first, functional fixedness, is the tendency to revert to an initial misconception. The second, innovation, is a drive to improve one's understanding. Functional fixedness initially increases as understanding departs from an initial conception and then decreases as a correct understanding is approached. Innovation is a monotonically decreasing function of U, greatest when understanding is far from the correct insight and decreasing as understanding approaches the correct insight. We modeled the continuous dynamics of understanding, U, using a functional form adapted from ecology [7, 8]:

$$\frac{dU}{dt} = \gamma \frac{(1-U)^2}{(0.1^2 + (1-U)^2)} - U(1-U) \tag{2}$$

The first term reflects the tendency to innovate. The second term reflects functional fixedness. The relative contribution of innovation is determined by the control parameter, γ. As we show below, our results are quite general and not specific to this functional form, which we chose because it is a well-studied model of metastable dynamics [7, 8] that generates some of the qualitative features of sudden insight.

We implemented this as a stochastic differential equation with additive Gaussian white noise:

$$dU_t = (\gamma \frac{(1-U_t)^2}{(0.1^2+(1-U_t)^2)} - U_t(1-U_t))dt + \sigma dW_t \quad (3)$$

where σ is a constant representing the magnitude of the noise. Thus, latent understanding U evolves under a regime of stochastic forcing, with infinitesimal "kicks" from the Wiener noise term σdW.

In the model, observable behavior is a symbolic Markov process, with the probability of shifting attention from one inscription to another determined by current understanding U_t. Each run was initialized with two random transition matrices, one that corresponded to complete confusion ($\mathbf{B}_0$) and one that corresponded to enlightenment ($\mathbf{B}_1$). The transition probabilities governing observable behavior for intermediate values of understanding were linearly interpolated between these extremes: $\mathbf{B}_U = (1-U)*\mathbf{B}_0 + U*\mathbf{B}_1$.

To correspond with the empirical dataset, we simulated $N = 24$ synthetic proof sessions, each with $N = 25$ inscriptions. Stochastic dynamics were simulated using Euler's method [9]. On each run, the control parameter γ increased linearly over time from 0.1 to 0.3. Both "confused" and "enlightened" attractors exist for $0.1 < \gamma < .26$, but the "confused" attractor disappears at the critical value $\gamma = 0.26$ (Supplementary Fig S1 a–d).

2 Supplemental Results

2.1 Coupled real-valued and symbolic dynamics: Analytic results

2.1.1 Setup

In the Main Text, we demonstrate numerically that, in our minimal model of insight and interaction, increased variability in the underlying continuous dynamics of understanding is indexed by increased surprisal in the observed symbolic time series. Here we explore analytically the circumstances under which surprisal in an 'observed' symbolic time series is an index of increased variability in an 'underlying' real-valued time series. (We use 'observed' and 'underlying' to evoke the scenario described in the Main Text, in which the observer only has access to the symbolic dynamics.)

Let $X(t)$ be a real-valued time series. Let $S(t)$ be a symbolic time series consisting of a sequence of symbols drawn from a finite library $\Omega = \{\omega_1, \omega_2, ...\}$. Assume these two time series are related such that each $x \sim X$ determines the probability of each symbol being emitted by the symbolic process, which we denote $P_x := P_x(\omega)$ for $\omega \in \Omega$. In other words, the 'observed' process S is a stochastic function of the 'underlying' real-valued process X.

We make an additional assumption about how S relates to the underlying values of X. We assume the mapping $x \mapsto P_x$ is affine, which intuitively captures the fact that changes in x should produce relatively well-behaved changes in P_x.

This assumption holds for a large class of mappings between 'underlying' real-valued dynamics and 'observable' symbolic dynamics — including the minimal model of insight described in the Main Text, where

the probability of an observed event (i.e., a transition from one inscription to another) is the linear combination of two anchor probability distributions, weighted by the latent real-valued understanding.

Here we explore when this assumption implies that, whenever the underlying real-valued time series $X_t = \{x_1, x_2, x_3, ..., x_t\}$ is more variable, the coupled symbolic time series $S_t = \{s_1, s_2, s_3, ..., s_t\}$ will on average be more unpredictable from its history (i.e., will have higher expected surprisal, given the observed symbols):

$$\mathrm{Var}(X) \uparrow \implies \mathbb{E}[h_{emp}(s_t)] \uparrow \tag{4}$$

where $h_{emp}(s_t)$ is surprisal, conditioned on the empirically observed symbols $S_t = \{s_1, s_2, s_3, ..., s_t\}$.

2.1.2 Argument

We define the expected surprisal of a new symbolic sample from P_x when evaluated under the empirical population as $g_t(x) := \mathbb{E}_{s\sim P_x}[-\log(P_{emp}(s))]$. We want know how $\mathbb{E}_{x_t\sim X}[g_t(x)]$ varies with $\mathrm{Var}(X)$

We first show that $P_{emp} \to P_\mu$ as the number of observations increases. Recall that P_{emp} is the empirical distribution over possible symbols Ω after t observations. Formally:

$$P_{emp}(s) := \frac{1}{t}\sum_{i=1}^{t} \delta_{s_i}(s), \text{where } \delta_{s_i}(s) = \begin{cases} 1 & s_i = s \\ 0 & s_i \neq s \end{cases}$$

Now define $P_{mix} := P_{mix}(s) := \mathbb{E}_{x\sim X}[P_x]$, the expected probability distribution over Ω if we draw randomly from X. By the law of large numbers, $P_{emp} \to P_{mix}$ as $t \to \infty$.

Thanks to the affine mapping $x \mapsto P_x$, and the linearity of expectation, this mixture distribution is simply the distribution associated with the mean of X, denoted μ. Thus, $P_{emp} \to P_\mu$. Thus, as the number of observations increases, the empirical distribution converges to a fixed distribution, P_μ, that depends only on the mean of X.

We next decompose the Expected Surprisal. Recall that we want to understand the behavior of the expected surprisal, which we denote ES. Expanding the definition of ES:

$$\begin{aligned} \mathrm{ES} &= \mathop{\mathbb{E}}_{x\sim X}[g_t(x)] \\ &= \mathop{\mathbb{E}}_{x\sim X}\left[\mathop{\mathbb{E}}_{s\sim P_x}[-\log(P_{emp}(s))]\right] \\ &= \mathop{\mathbb{E}}_{x\sim X}\left[\sum_{\omega\in\Omega} P_{x_t}(\omega)(-\log(P_{emp}(\omega)))\right] \end{aligned}$$

The inner expectation is the cross-entropy between the true distribution at time t, P_{x_t}, and the empirical one, P_{emp}. Since expectations preserve limits under bounded convergence, as $P_{emp} \to P_\mu$, the cross-entropy $\mathrm{CE}(P_{x_t}, P_{emp})$ converges to $\mathrm{CE}(P_{x_t}, P_\mu)$.

Using the standard decomposition of cross-entropy:

$$\mathrm{CE}(P_{x_t}, P_\mu) = H(P_{x_t}) + D_{KL}(P_{x_t} \| P_\mu)$$

where H is the Shannon entropy and D_{KL} is the Kullback–Leibler divergence. By linearity of expectations we have:

$$\mathrm{ES} = \mathop{\mathbb{E}}_{x\sim X}[H(P_{x_t})] + \mathop{\mathbb{E}}_{x\sim X}[D_{KL}(P_{x_t} \| P_\mu)] \tag{5}$$

Finally, we exploit convexity to prove our target claim. To do so we isolate the term that grows with increased variance, the KL-divergence term. By a standard result, if $x \mapsto P_x$ is affine, then $D_{KL}(P_x, Q)$ is a convex function of x for any fixed distribution Q. In our case, $P_{emp} \to P_{mix} = P_\mu$, and $x \mapsto P_x$ is affine by assumption, so $D_{KL}(P_x, P_emp)$ is convex for large t. By Jensen's inequality, wherever f is a convex function, then a mean-preserving spread of X (i.e., a distribution with the same mean as but a larger variance than X) implies a non-decreasing expectation of f. If f is strictly convex, then the expectation is strictly increasing. Thus, since D_{KL} is strictly convex, for non-degenerate cases, if we increase the variance of X but maintain its mean, then the expectation of the D_{KL} term increases:

$$\mathrm{Var}(X) \uparrow \implies \mathbb{E}[D_{KL}(P_{x_t} \| P_{emp})] \uparrow \tag{6}$$

Finally, we can analyze the effect of variance on the two terms in Equation 5. By Jensen's inequality, an increase in $Var(X)$ has two opposing effects. First, because KL-divergence is a convex function of P_x (and thus x), a mean-preserving increase in the variance of X increaes the expected KL-divergence term, $\mathbb{E}[D_{KL}(P_x \| P_\mu)]$. Second, because Shannon entropy H is a concave function, the same increase in variance decreases the expected entropy term, $\mathbb{E}[H(P_x)]$.

Therefore, our central claim that $\mathrm{Var}(X) \uparrow$ implies $\mathbb{E}[h_{emp}(s_t)] \uparrow$ holds precisely when the first effect dominates the second. While this condition is not guaranteed to hold universally, it provides a compelling explanation for the numerical results in our Main Text. Assuming this dominance holds for our model, we arrive at our final conclusion:

$$\mathrm{Var}(X) \uparrow \implies \mathbb{E}[h_{emp}(s_t)] \uparrow \tag{7}$$

In the Main Text, we show that (7) holds numerically for our minimal model when we use a window that is analogous in size to the window used in our empirical analyses.

2.2 Empirical results with the addition of pilot proof sessions

The empirical data analyses of the Main Text include 12 proof sessions (3 men and 3 women) and a total of 24 insights across 11 sessions (i.e., one session did not include a verbal expression of an insight). The pilot data consisting of a male mathematician, with 3 insights across 2 proof sessions was excluded from the statistical analysis of the Main Text. Here, we show that results remain unchanged when including this additional data (Fig. S2).

We constructed a linear mixed effects model of the surprisal of each shift of attention. This model included fixed effects for time, for whether the event was in the period before the flash of insight, for whether the event was in the period after the flash of insight, and for the interaction between these latter two. We added a fixed effect for whether the event involved introducing an entirely new inscription, since these would have higher surprisal by definition. We also included random intercepts for sessions.

Surprisal increased over the course of the session, with a .003 increase in surprisal for every passing minute ($b = .003 \pm .002$ SE, $t = 1.57$, $p = .11$). Unsurprisingly, shifts of attention to entirely new inscriptions had significantly higher surprisal ($b = .954 \pm .06$ SE, $t = 14.87$, $p < .001$). Critically, surprisal was significantly greater in the one-minute periods both immediately before the flash of insight ($b = .14 \pm .05$ SE, $t = 2.61$, $p < .01$) and immediately after the flash of insight ($b = .19 \pm .05$ SE, $t = 3.64$, $p < .001$). The interaction was not significant ($b = -.16 \pm .11$ SE, $t = -1.39$, $p = .16$).

2.3 Window size sensitivity analysis for the empirical results

In the Main Text, we reported empirical results based on a fixed context window of 60 seconds. We conducted a sensitivity analysis of the duration of this context window for values +/- 33% of the value used in the Main Text. This analysis confirmed that our results are robust to variations in the size of the context window (Fig. S3).

3 Supplemental Figures

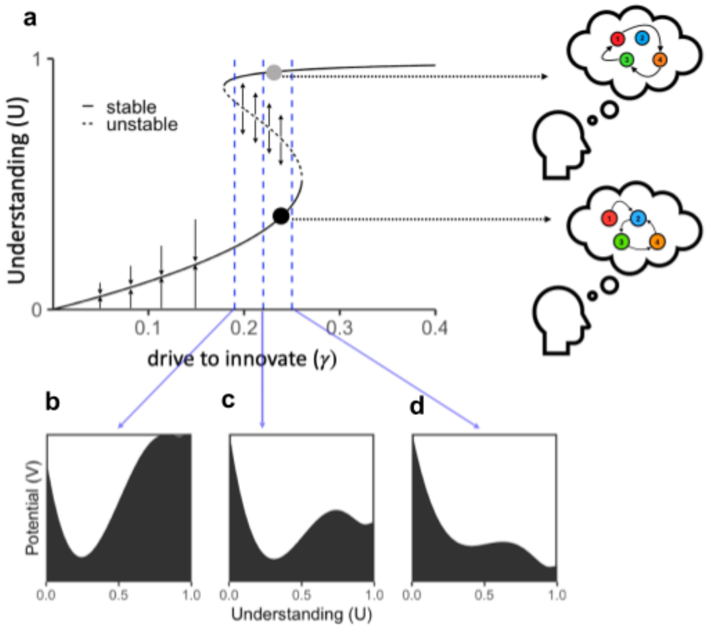

Figure S1. Moment-to-moment understanding can be conceptualized as a dynamical trajectory in a landscape of ideas. (a) The individual's state of mind is manifested as a network of connections between ideas at every moment. The vertical axis represents the mental state, the horizontal axis represents the control parameter, drive to innovate (γ). The S shape curve shows the attractors of the system for different values of the control parameter. The misconception attractor is marked with a black circle, and the insight attractor with a gray circle. As shown on the right side, each alternative state (misconception or insight) is characterized by a unique pattern of connections across ideas. The numbered nodes mark ideas; arrows mark connections between ideas. The solid arrows indicate the direction of change. In this case, equilibria on the dashed middle section of the S curve are unstable, representing the border between the basins of attraction for two alternative stable states. The blue dashed lines represent three values of the control parameter and their corresponding state.

(b-d) Represent dynamics of understanding over time. The vertical axis represents potential energy; the horizontal axis represents understanding. (b) When the control parameter γ is below a critical value ($\gamma_{c1} \approx 0.18$), the hypothetical mathematician is drawn back to the misconception state (i.e., U close to 0). In this case, a single attractor — representing the misconception state — appears in the landscape. (c) For intermediate values of the control parameter ($\gamma_{c1} < \gamma < \gamma_{c2}$), two stable attractors appear in the landscape: one for insight and one for misconception. (d) When the control parameter is greater than a second critical value ($\gamma_{c2} \approx 0.26$), understanding grows to an attractor slightly less than 1 (i.e., the insight attractor).

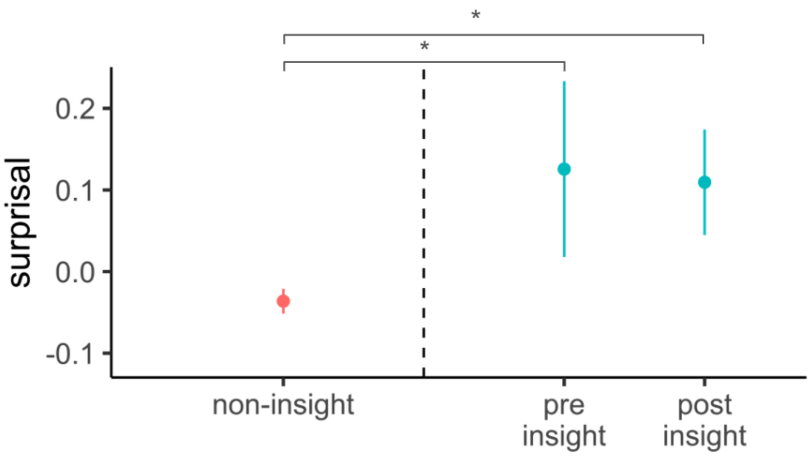

Figure S2. Sudden insights among real-world mathematicians were anticipated by increased unpredictability of their blackboard interactions, with pilot data included. We confirmed that the results of the Main Text were unchanged when the data of a pilot mathematician was included. Mathematicians made more unexpected connections during an insight. The vertical axis represents the surprisal of shifts of attention between inscriptions (z-scored within individuals to account for individual differences). *Non-insight* shifts of attention, in red, did not occur near a flash of insight. Insight, in teal, was divided into *pre-insight* (the period immediately before the flash of insight) and *post-insight* (the period immediately after). Dots indicate means; error bars indicate SEM (* indicates $p < .05$).

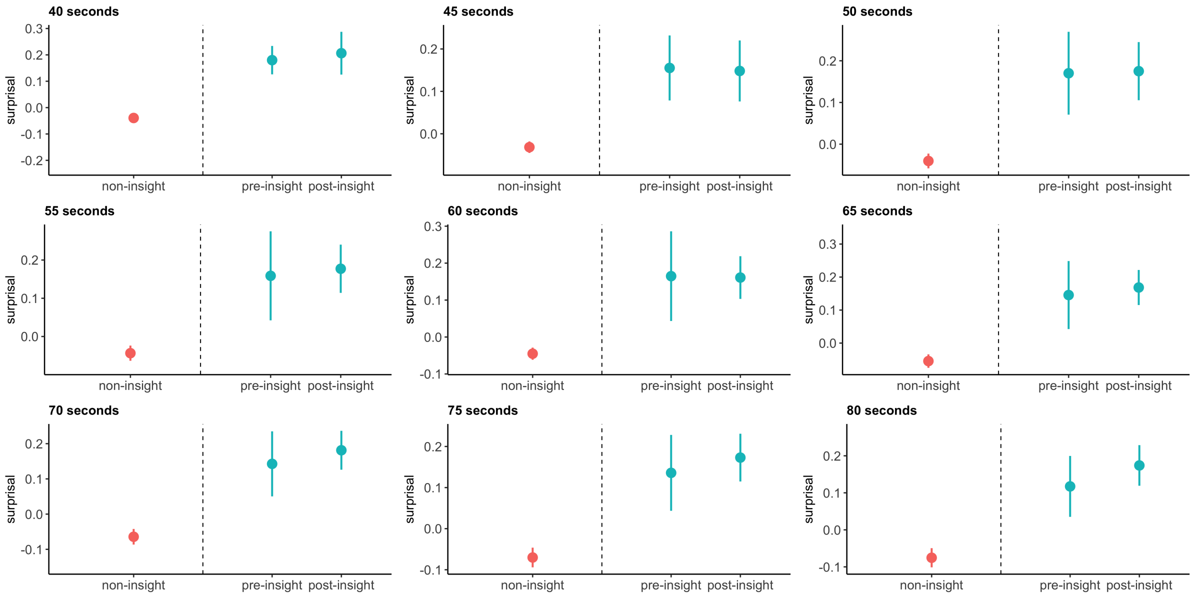

Figure S3. Sensitivity analysis of the width of the context window used to calculate surprisal. Surprisal for each event was calculated using a context window of width δ. In the Main Text, we set $\delta = 60$ seconds. Here we varied the width from 40 to 80 seconds (±33% of 60 seconds). Each panel shows the main result for a given value of δ. To compare across values of δ, we standardized (i.e., z-scored) the values of surprisal. The results were consistent across context widths: events were increasingly unpredictable around the time of insight (pre- and post-insight; teal dots) compared to other events (non-insight; red dots). (Dots = means; error bars = standard errors).

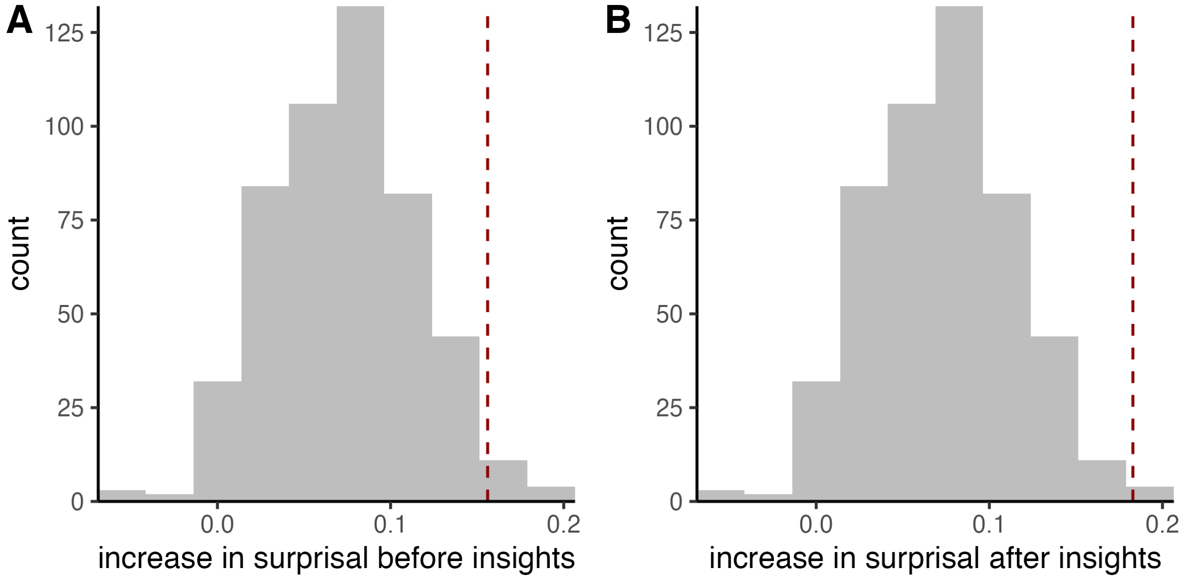

Figure S4. Time-permuted null model of blackboard interactions. We confirmed the robustness of the observed increase in surprisal in the vicinity of insights by comparing this observed increase to a time-permuted null model. We generated time-permuted surrogate time series ($N = 500$) by randomly shuffling the timing of blackboard interactions within each proof session. This preserves the number of inscriptions, the number of interactions with each inscription, and the timing of when mathematicians interacted with inscriptions, but randomizes which inscription is interacted with at each moment. For each surrogate time series, we calculated the increase in surprisal in the vicinity of insights. **(A)** Null distribution of the increase in surprisal in the period immediately before an insight. The dashed vertical line indicates the empirically observed increase, which was reliably greater than expected under this null ($p = .03$). **(B)** Null distribution of the increase in surprisal in the period immediately after an insight. The dashed vertical line indicates the empirically observed increase, which was reliably greater than expected under this null ($p < .01$).

References

1. Marghetis, T., Samson, K. & Landy, D. The complex system of mathematical creativity: Modularity, burstiness, and the network structure of how experts use inscriptions. *CogSci* 763–769 (2019).

2. Wittenburg, P., Brugman, H., Russel, A., Klassmann, A. & Sloetjes, H. Elan: A professional framework for multimodality research. In *5th international conference on language resources and evaluation (LREC 2006)*, 1556–1559 (2006).

3. Lausberg, H. & Sloetjes, H. Coding gestural behavior with the neuroges-elan system. *Behav. research methods* **41**, 841–849 (2009).

4. R Core Team. *R: A Language and Environment for Statistical Computing*. R Foundation for Statistical Computing, Vienna, Austria (2024).

5. Kuznetsova, A., Brockhoff, P. B. & Christensen, R. H. B. lmertest package: tests in linear mixed effects models. *J. statistical software* **82**, 1–26 (2017).

6. Wallas, G. *The Art of Thought* (Brace and Co, New York: Harcourt, 1926).

7. Ludwig, D., Jones, D. D., Holling, C. S. *et al.* Qualitative analysis of insect outbreak systems: the spruce budworm and forest. *J. Animal Ecol.* **47**, 315–332 (1978).

8. May, R. M. Thresholds and breakpoints in ecosystems with a multiplicity of stable states. *Nature* **269**, 471–477 (1977).

9. Protter, P. E. & Protter, P. E. *Stochastic differential equations* (Springer, 2005).
